# Supplementary material for: A Longitudinal Seroprevalence Study Evaluating Infection Control and Prevention Strategies at a Large Tertiary Care Center with Low COVID-19 Incidence
Source: Int J Environ Res Public Health. 2021 Apr 15;18(8):4201. doi: 10.3390/ijerph18084201 (PMC8071361; doi:10.3390/ijerph18084201)
Supplement: Supplementary file 1 [file ijerph-18-04201-s001.zip › ijerph-1160491-supplementary.pdf]

Supplementary material

Evaluation of infection control and prevention strategies, a longitudinal seroprevalence study at a large tertiary care center with low COVID-19 incidence.

Supplementary table S1 highlights the COVID-19 control strategies implemented by the Austrian government to reduce daily infection rate. COVID-19, Coronavirus Disease 19;

**Implemented COVID-19 control strategies by the Austrian government initiated on March 16th**
1. Access to public areas was restricted[a]
2. Stores were closed unless essential (pharmacies, grocery stores, etc.)[b]
3. Teaching operations on universities and schools were transitioned to home schooling and home office[c]
4. Prohibited entering of supermarkets or public transport without medical masks[a]
5. Restricted entrance into the country[a]

[a] Ongoing throughout the whole study period [b] Gradual loosening of control strategies, with opening of small stores (April 14th), larger stores and hairdressers (>400m^2), opening of gastronomy (May 15th), hotels and fitness centers (May 29th). [c] Gradual loosening of restrictions in school and university from May 4th to June 3rd.

Scheme 2. highlights implemented infection prevention strategies at the General Hospital Vienna. Implemented measures were ongoing throughout the study period. COVID-19, Coronavirus Disease 19; HCWs, Health Care Workers; FFP, Filtering Face Piece; WHO, World Health Organization.

**Implemented COVID-19 control strategies initiated on March 19th**
1. Formation of distinct COVID-19 wards
2. Obligatory medical masks for all HCWs
3. Compulsory screening for patients admitted to our hospital
4. Personal protective equipment (consisting of FFP2 masks [FFP3 for aerosol generated procedures], goggles or face shields, long-sleeved water resistant gowns and a minimum of one pair of gloves) for HCWs in contact with COVID-19 patients
5. Quarantine for positively tested HCWs and HCWs with history of high-risk COVID-19 contact[a]
6. Hand hygiene according to recommendations of the WHO (23)

Supplementary table S3 summarizes the different wards participated at this study and highlights their distribution to Table 19. Coronavirus Disease 2019; OPD, outpatient department.

[b] High-risk contact was defined as an unprotected contact to COVID-19 patients for a period of ≥15 minutes within ≤2 meters of rang	COVID-19 OPD	COVID-19 ward	COVID-ICU	Internal medicine ward	Internal medicine OPT	Operation room	ICU (adults and children)	Surgical OPT	Surgical Ward	Neurological ward	Neurological OPT	Paediatric OPT	Paediatric ward	Dental clinic	Laboratory	Radiology	Pathology	Office	Overall
Physician	28	11	18	17	21	2	9	19	13	4	3	7	8	23	0	1	6	0	190
Nurse	18	57	39	37	13	29	11	25	8	2	2	1	20	10	0	0	0	0	272
Secretary	0	3	0	6	7	0	0	5	0	0	0	0	0	1	0	0	0	1	23
Administrative staff	4	4	6	6	8	0	2	3	0	0	0	0	1	0	0	0	0	0	34
Student	5	2	2	5	0	0	0	0	0	0	0	0	0	0	1	0	0	0	15

voluntary worker	0	2	0	1	0	0	0	0	0	0	0	0	0	0	0	0	0	0	3
MTA	1	0	0	1	2	0	0	7	0	0	0	0	1	0	0	5	8	0	25
Physiotherapist	0	0	3	3	0	0	1	0	0	0	0	0	1	0	0	0	0	0	8
Psychologist	0	0	0	1	0	0	0	0	0	0	1	1	2	0	0	0	0	0	5
non-clinical woker	0	0	2	7	8	0	0	1	0	0	0	0	0	0	2	0	2	2	24
not reported	0	0	0	2	0	0	0	0	0	0	0	0	0	0	0	0	0	0	2
Over all	56	79	70	86	59	31	23	60	21	6	6	9	33	34	3	6	16	3	599
**Cohorts**	**COVID-19 OPD**	**COVID-19 ward**	**COVID-19 ICU**	**Normal ward**	**OPD**	**Normal ward**	**ICU**	**OPD**	**Normal ward**	**Normal ward**	**OPD**	**OPD**	**Normal ward**	**OPD**	**Normal ward**	**Normal ward**	**Normal ward**	**Normal ward**	

Scheme 4. highlights areas at risk for COVID-19 in March 2020.

Country	Areas	start date
Egypt	whole country	05.03.2020
China	whole country	04.01.2020
South Korea	whole country	19.01.2020
Iran	whole country	19.02.2020
Japan	whole country	25.01.2020
Singapore	whole country	23.01.2020
USA	Washington, New York and New Jersey	20.01.2020
Italy	whole country	29.01.2020
Great Britain	London	01.02.2020
Spain	Madrid, Navarre, La Rioja and Pais Vasco	10.03.2020
Germany	North Rhine-Westphalia	28.01.2020
France	Grand Est and Ile-de-France	24.01.2020
Switzerland	Tessin, Waadt and Geneva	24.02.2020
Austria	Tyrol (whole), Vorarlberg (Lech, Warth, Schröcken, Klösterl, Nenzing-Dorf and Beschling), Carinthia (Heiligenblut), Salzburg (Flachau, Bad Gastein, Bad Hofgastein, Dorfgastein, Großarl and Hüttschlag)	26.02.2020

Scheme 5. highlights the odds for COVID-19 depending on symptoms.

Symptom	Tested negative	Tested positive	Odds ratio	95% CI	p value
Fever	7.1%	33.3%	6.57	1.17-36.92	0.033
Headache	12.5%	33.3%	3.51	0.63-19.52	0.151
Cough	12.0%	50.0%	7.37	1.46-37.20	0.016
SoreThroat	14.3%	0.0%			
Sneezing	7.4%	0.0%			
BodyAches	5.9%	16.7%	3.19	0.36-28.09	0.295
Sniffing	12.1%	66.7%	14.50	2.61-80.59	0.002
Diarrhea	5.1%	16.7%	3.76	0.43-33.20	0.233
ShortnBreath	3.2%	16.7%	6.05	0.67-54.36	0.108
Smell	1.9%	50.0%	53.00	9.61-292.41	<0.001
Fatigue	12.3%	50.0%	7.14	1.41-36.03	0.017

Supplementary table S6 comprises the different cohorts and highlights positive tested health care workers, COVID-19, Coronavirus Disease 19; ICU, intensive care unit;.

Working environment	Tested positive depending on working environment
**COVID-19 ICU** (n=70)	**-** tested positive =1
**ICU** (n=29)	- ICU – adults (n=23, tested positive 0)<br>- ICU – children (n=6, tested positive 0)

**COVID-19 ward** (n=79)	- tested positive = 1
**Normal ward** (n=199)	- Internal medicine (n=86, tested positive 1)<br>- Surgical department (n=21, tested positive 0)<br>- Operating room (n=31, tested positive 0)<br>- Neurological ward (n=6, tested positive 0)<br>- Pathology department (n=16, tested positive 0)<br>- Radiology department (n=6, tested positive 0)<br>- Paediatric department (n=27, tested positive 0)<br>- Office(n=3, tested positive 0)<br>- Laboratory (n=3, tested positive 0)
**COVID-19 outpatient department (**n=56)	- tested positive=2
**Outpatient department** (n=166)	- Internal medicine (n=57, tested positive 0)<br>- Surgical department (n=60, tested positive 2)<br>- Paediatric department (n=9, tested positive 0)<br>- Dental clinic (n=34, tested positive 0)<br>- Neurological department (n=6, tested positive 1)

Supplementary table S7 highlights risk factors assessed in our study. Proportion is shown as n,(%). The different proportion of risk factors was further calculated by Fisher's exact probability tests. Significant differences (p<0.05) were highlighted by *.

**Risk factors**	**negatively tested HCW (n=591)**	**positively tested HCW (n=8)**	**p-value**
Working environment	201 (34%)	4 (50%)	0.455
Unprotected contact	47 (8%)	4 (50%)	0.003*
Unprotected contact in work	42 (7.1%)	2 (25%)	0.11
Unprotected contact in leisure	5 (0.8%)	2 (25%)	0.003*
Family history			
Family member in quarantine	30 (5%)	2 (25%)	0.06
Family member tested positive	2 (0.3%)	1 (12.5%)	0.04*
Travel history	37 (6.3%)	2 (25%)	0.09
Problems handling PPE	30 (5%)	0	1

Supplementary figures:

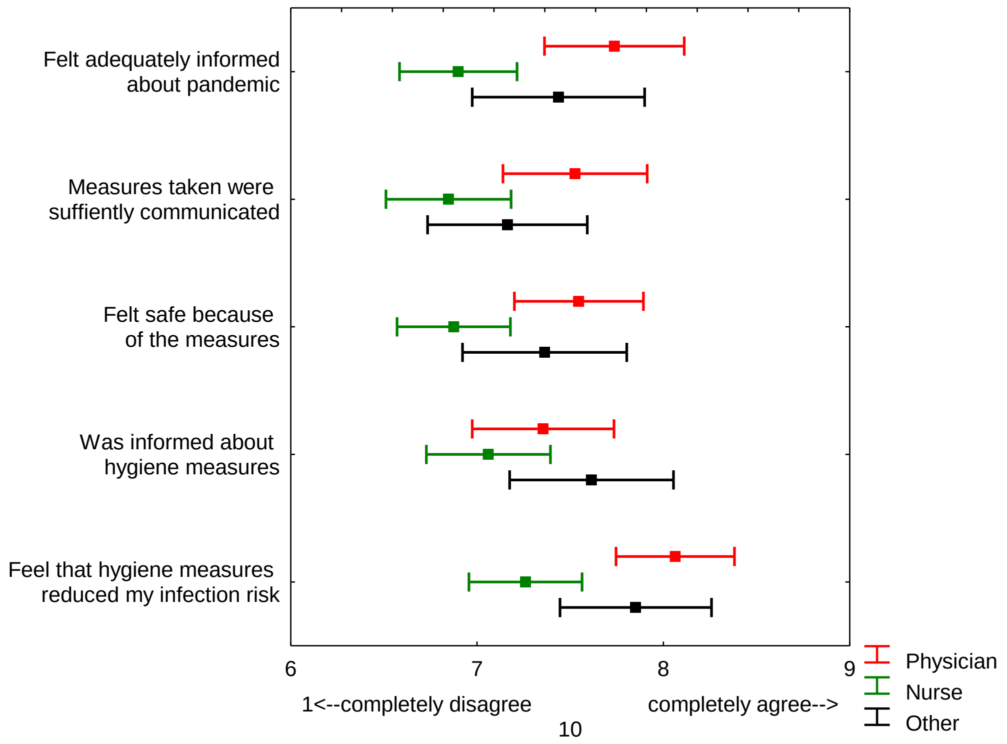

Scheme 8. demonstrates how the HCWs have experienced the epidemic at our center.

Questionnaires (translated from German):

Questionnaire: A cross-sectional study assessing SARS-CoV-2 seroprevalence at the Vienna General Hospital during COVID-19 epidemic.

A) Subject Code: ________________________________

1. Demographic parameters

1.1. Sex ❐ male ❐ female

1.2. Do you smoke? ❐ no ❐ yes ❐ former smoker

1.2.1. When have you started smoking? ___/___/_______

1.2.2. When did you stop smoking? ___/___/_______

1.2.3. How many cigarettes per day? ______cigarettes/day

2. Risk profile

2.1. Working environment

2.1.1. Outpatient wards

❐ COVID-19 outpatient ward ❐ Other:_____________

2.1.2. Inpatient wards

❐ COVID-19 ward ❐ ward

❐ COVID-19 intermediate care unit ❐ intermediate care unit

❐ COVID-19 intensive care unit ❐ intensive care unit

❐ ward for suspected COVID-19 cases ❐ day clinic

❐ other: ____

2.1.3. Profession

❐ Physician ❐ Nurse ❐ Administrative staff

❐ Housekeeping ❐ Student ❐ Voluntary worker

❐ Others:____

2.1.4. Since when have you been working at the mentioned ward? (DD/MM/JJJJ) ___/___/_______

2.1.5. Have you had contact to a COVID-19 patient? ❐ yes ❐ no

2.1.5.1. When did the first contact take place? (DD/MM/JJJJ) ___/___/_______

2.1.6. How often have you had contact to a COVID-19 patient within the last two weeks?

❐ none ❐ once ❐ once a week

❐ 2-6 times per week ❐ once a day ❐ several times a day

2.1.7. Do you work at other wards as well?

❐ COVID-19 ward ❐ Ward

❐ COVID-19 intermediate care unit ❐ intermediate care unit

❐ COVID-19 intensive care unit ❐ intensive care unit

❐ ward for suspected COVID-19 cases ❐ day clinic

❐ Other:____

2.2. Travel history

2.2.1. Have you been outside Austria within the last 6 months? ❐ yes ❐ no

2.2.1.1. If you answered question 2.2.1 with yes, please state where you have been to.

Country	City	Date of arrival	Date of departure
1) _______________	______________	_______________	_______________
2) _______________	______________	_______________	_______________
3) _______________	______________	_______________	_______________

2.2.2. Have you been outside Vienna within the last 6 weeks? ❒ yes ❒ no

2.2.2.1. If you answered question 2.2.2. with yes, please state where you have been to.

Country	City	Date of arrival	Date of departure
1) _______________	______________	_______________	_______________
2) _______________	______________	_______________	_______________
3) _______________	______________	_______________	_______________

2.3. Living condition

2.3.1. Do you live in Vienna? ❒ yes ❒ no

2.3.1.1. If you answered question 2.3.1. with no, please state where you live.

❒ Lower Austria ❒ Burgenland ❒ Upper Austria

❒ Styria ❒ Carinthia ❒ Tyrol

❒ Salzburg ❒ Vorarlberg

City/Town: ____

2.3.2. With how many people do you live in the same household? ______Number

2.3.3. How many children under 10 years live with you? ______Number

2.3.4. How many children above 10 years live with you? ______Number

2.3.5. How many adults above 65 years live with you? ______Number

2.3.6. Did one of your roommates have to go in to quarantine? ❒ yes ❒ no

2.3.6.1. Did the person in quarantine report symptoms? ❒ yes ❒ no

2.3.6.2. Was the person in quarantine positively tested for SARS-CoV-2? ❒ yes ❒ no

2.3.6.3. How often have you had contact to the person in quarantine?

❒ once ❒ once a week ❒ 2-6 times per week

❒ once a day ❒ several times per day

2.3.7. Have you had contact to COVID-19 outside of your family or work? ❒ yes ❒ no

2.3.7.1. When did the COVID-19 contact take place? (DD/MM/YYYY) ___/____/______

2.3.8. How do you get to work? (please add percentage if more than one mean of transport is used)

❒ Tram ___% ❒ Subway ___% ❒ Car/Motorcycle ___%

❒ Train ___% ❒ Bus ___% ❒ Bicycle ___%

❒ By foot ___% ❒ Others:________________________ ___%

3. Personal protective equipment

3.1. Personal protective equipment during work in contact with COVID-19 patients

3.1.1. Have you been protected by personal protective equipment during COVID-19 contact? (Personal protective equipment consisting of FFP2 masks [FFP3 for aerosol generated procedures], goggles or face shields, long-sleeved water resistant gowns and a minimum of one pair of gloves)

❒ always (~100%) ❒ often (~80%) ❒ sometimes (~50%) ❒ never

3.1.2. If not protected according to point 3.1.1., how were you protected during COVID-19 contact? (please select all options that apply)

❒ goggles/face shield ❒ long-sleeved water-resistant gown

❒ gloves ❒ FFP 2 masks

❒ others:____

3.2. Personal protective equipment during leisure time in contact with COVID-19 patients

3.2.1. How often have you been protected by personal protective equipment during COVID-19 contact in leisure time?

❒ always (~100%) ❒ often (~80%) ❒ sometimes (~50%) ❒ never

3.2.2. How were you protected during COVID-19 contact in leisure time? (please select all options that apply)

❒ Gloves ❒ FFP2 masks

❒ Others:____

3.3. Have you had problems in handling the personal protective equipment? ❒ yes ❒ no

3.3.1. If you have reported problems in handling personal protective equipment, please specify the kind of problems that have occurred:

____

____

____

____

3.4. How much does the following statement apply? (1 does not apply, 10 applies fully)

	1	2	3	4	5	6	7	8	9	10
I felt protected by personal protective equipment	❒	❒	❒	❒	❒	❒	❒	❒	❒	❒

3.5. Have you had unprotected contact to a COVID-19 positive individual? ❒ yes ❒ no

3.5.1. Where did the unprotected contact to a COVID-19-positive individual take place?

❒ Work ❒ Leisure time ❒ Work and leisure time

4. Quarantine

4.1. Have you had to go into quarantine? ❒ yes ❒ no

4.1.2. If you had to go into quarantine, please state the date and reason for said quarantine.

	Start date	End date	Reason
1. Quarantine	___/ _____/ ______	___/ _____/ ______	❐ Stay in a risk-area<br>❐ Contact to COVID-19-positive individual<br>❐ COVID-19 disease<br>Other:________________
2. Quarantine	___/ _____/ ______	___/ _____/ ______	❐ Stay in a risk-area<br>❐ Contact to COVID-19-positive individual<br>❐ COVID-19 disease<br>Other:_________________

5. History of SARS-CoV-2 tests

5.1. Have you been tested for SARS-CoV-2? ❐ yes ❐ no

5.1.1. When did you get tested for SARS-CoV-2 and what was the result?

**Tests**	**Date (DD/MM/YYYY)**	**Result (positive/negative)**
1. Test	_____/_____/________	______________________
2. Test	_____/_____/________	______________________
3. Test	_____/_____/________	______________________
4. Test	_____/_____/________	______________________
5. Test	_____/_____/________	______________________
6. Test	_____/_____/________	______________________
7. Test	_____/_____/________	______________________
8. Test	_____/_____/________	______________________
9. Test	_____/_____/________	______________________
10. Test	_____/_____/________	______________________

6. Symptoms

6.1.	Have you been ill since 25.02.2020?	❐ yes	❐ no
6.2.	If you answered 6.1. with yes, please answer the following questions.		
6.2.1.	First episode of illness:		
6.2.1.1	When did the symptoms start? (DD/MM/JJJJ)	__/ ___/ _____	
6.2.1.2.	When did the symptoms end? (DD/MM/JJJJ	__/ ___/ _____	
6.2.1.3.	What kind of symptoms did you experience?		

❐ Fever	❐ Headache	❐ Cough	❐ Sore throat
❐ Sneezing	❐ Limb pain	❐ Congestion or runny nose	❐ Diarrhoea
❐ Fatigue	❐ Shortness of breath	❐ Altered taste or smell	

❒ Other: ____

6.2.2. Second episode of illness:

6.2.2.1 When did the symptoms start? (DD/MM/JJJJ) __/ ___/ _____

6.2.2.2. When did the symptoms end? (DD/MM/JJJJ __/ ___/ _____

6.2.2.3. What kind of symptoms did you experience?

❒ Fever	❒ Headache	❒ Cough	❒ Sore throat
❒ Sneezing	❒ Limb pain	❒ Congestion or runny nose	❒ Diarrhoea
❒ Fatigue	❒ Shortness of breath	❒ Altered taste or smell	

❒ Other: ____

7. Experience of the COVID-19 epidemic at the General Hospital Vienna

How much does the following statement apply? (1 does not apply, 10 applies fully)

	**1**	**2**	**3**	**4**	5	**6**	**7**	**8**	**9**	**10**
I felt adequately informed about the epidemic.	❒	❒	❒	❒	❒	❒	❒	❒	❒	❒
Measures taken were sufficiently communicated.	❒	❒	❒	❒	❒	❒	❒	❒	❒	❒
The measures made me feel safe.	❒	❒	❒	❒	❒	❒	❒	❒	❒	❒
I was informed about hygiene measures.	❒	❒	❒	❒	❒	❒	❒	❒	❒	❒
I felt that hygiene measures reduced my infection risk.	❒	❒	❒	❒	❒	❒	❒	❒	❒	❒
